# Overcoming difficulties in segmentation of hyperspectral plant images with small projection areas using machine learning

**DOI:** 10.1038/s41598-025-31952-9

**Published:** 2026-01-30

**Authors:** Eva Neuwirthová, Jiří Chuchlík, Miroslav Pikl, Zuzana Lhotáková, Ivan Kashkan, Klára Panzarová, Jan Stejskal, Jana Albrechtová, Milan Lstibůrek, Jaroslav Čepl

**Affiliations:** 1 Department of Forest Genetics and Physiology, Faculty of Forestry and Wood Sciences, Czech University of Life Sciences, Prague, Czech Republic; 2 Department of Experimental Plant Biology, Faculty of Science, Charles University, Prague, Czech Republic; 3 Global Change Research Institute of the Czech Academy of Sciences, Brno, Czech Republic; 4 Photon Systems Instruments, (PSI, s.r.o.), Drásov, Czech Republic; 5 Laboratory of Hormonal Regulations in Plants, Institute of Experimental Botany, Czech Academy of Sciences, Prague, Czech Republic

**Keywords:** Conifers, Phenotyping, Needle segmentation, Controlled environment, Water stress, Computational biology and bioinformatics, Ecology, Ecology, Plant sciences

## Abstract

Segmentation of hyperspectral image data is a well-established technique in remote sensing. While it is commonly applied to individual field crops, its use for individual trees is less prevalent. Conifers are crucial in forestry, and assessing physiological status, or genetic diversity is required for effective early-age treatment in nurseries and hyperspectral imaging (HSI) combined with high-throughput phenotyping (HTP) offers faster and non-destructive evaluation. NDVI-based thresholding is sufficient for detection of leaves with large projection areas, but needles of conifers present challenges due to spatial resolution constraints and increased proportion of border pixels. This study monitored the offspring of three locally adapted Scots pine (*Pinus sylvestris* L.) populations, representing distinct upland and lowland ecotypes. This study presents a hyperspectral image processing pipeline for segmenting and isolating individual Scots pine seedlings. Using a K-means algorithm, 23 hyperspectral centroids were successfully derived and subsequently classified into ten biologically distinct groups. Random forest classification model effectively differentiated Scots pine seedlings based on origin during water stress and recovery periods. This study highlights the potential of hyperspectral imaging and machine learning in evaluating the physiological state of conifer seedlings, demonstrating promising applications in forest tree physiology research and tree breeding.

## Introduction

In forestry, Scots pine (*Pinus sylvestris* L.), a widely distributed pioneer species^1^ known for its drought tolerance^2^, serves as a key model for studying adaptation and resilience^3^ to changing climatic conditions^4^. However, evaluating physiological traits in conifer seedlings presents unique challenges due to their complex morphology, particularly the segmentation of needle projection areas in imaging datasets.

This issue becomes even more critical when employing advanced technologies like hyperspectral imaging (HSI), which relies on precise extraction of spectral and spatial information to analyze small plant projection areas. HSI integrates spectral information across multiple wavelengths with imaging technology, forming a hyper-cube that captures both spectral and spatial characteristics of the object. However, the link between optical properties and physiological status is complex, and interpreting these signals often requires careful consideration of underlying biological processes^5^. HSI has achieved significant success in broader forestry applications, including identifying pathogens^6–10^, monitoring pollution-induced stress^11–14^, tracking phenological development^15^ and distinguishing provenances^16,17^. Additionally, it has been utilized at the individual seedling level for tasks such as disease detection^18,19^, chlorophyll and water content estimation^20^, phenotyping stress-tolerant traits^5,21,22^, and assessing the spatial distribution of needle chemical composition^23^. HSI has succesfully identified genetic differences in reflectance across evergreen and deciduous species^16,17,24^ as well as within Scots pine^25,26^.

Vegetation indices are frequently used for HSI segmentation, an approach that is particularly effective for plant organs and canopies with substantial projection areas^27,28^. These multispectral indices can capture phenology dynamics, genetic differentiation and climate adaptation patterns at the canopy scale in conifer trees^15,29^. However, in conifer seedlings, with regard to needle projection area, HSI requires a sophisticated and precise segmentation approach, as presented for pine^19,22,23^ and Chinese fir seedlings by^20^. However, the challenging segmentation of small plant projection areas obstructs its utility for distinguishing specific features such as ecotypic variation in conifer seedlings.

High-throughput phenotyping (HTP) enables rapid, non-destructive measurement of plant physiological traits using automated optical sensors. This approach has already proven effective in our previous work for detecting drought-induced variation in Scots pine seedlings, demonstrating its relevance and potential for advanced early-age phenotyping in conifers^30^. Despite the promise of HTP systems for efficient trait assessment^31–33^; their integration with HSI for individual seedlings evaluation remains underdeveloped. Accurate segmentation is particularly important for precise monitoring of water stress and recovery, where rich spectral data can reveal critical physiological and adaptive traits. However, there is still a lack of robust methodologies specifically designed for the segmentation of individual conifer seedlings. This methodological gap is particularly critical given the small surface area of needles and the complex three-dimensional architecture of conifer seedlings.

In this study, a question related to the use of HSI in Scots pine seedlings, has been addressed: Can hyperspectral imaging (HSI) accurately classify ecotypic variation in Scots pine seedlings during periods of water stress and recovery? To address the question, this study focuses on developing a robust HSI-based pipeline for Scots pine seedling analysis, which tackles the segmentation problem as a primary goal. In the current study, we aimed to differentiate among the three populations rather than to test pairwise differences, as was done by Stejskal et al.^25^.

Furthermore, the accurate segmentation of small projection areas is expected to enhance the ability to detect drought stress and to differentiate among locally adapted populations. Thus, we developed a classification model to account for the biological significance of each spectral cluster to discriminate Scots pine seedlings based on their progeny origin and physiological response to drought stress. By integrating HSI with machine learning and HTP, this study introduces a novel approach to addressing the critical segmentation problem, thereby facilitating the use of advanced imaging techniques in forest ecophysiology and tree improvement programs.

## Materials and methods

### Experimental design

Seed material comprised progeny from three seed orchards in the Czech Republic, representing lowland and upland Scots pine ecotypes. The lowland ecotypes originated from the Plasy orchard (49°90′87″N, 13°44′27″E) and the Trebon orchard (49°01′66″N, 14°82′47″E), while the upland ecotype originated from the Decin orchard (50°81′91″N, 14°11′72″E). The sample sizes included 303 and 299 individuals from the Plasy and Trebon orchards, respectively, and 189 individuals from the Decin orchard. Two years old seedlings from these three seed orchards represented three locally adapted populations with distinct ecological and geographical origins^34^. During the first growing season, the seedlings were cultivated in a common environment of planters in a nursery, for details, see^25^. In the following year during February (before the beginning of the 2nd growing season) seedlings were transplanted into 270 5-litre pots with three individuals representing one of the three populations per pot. Since fewer seedlings from the seed orchard in Decin were available, some pots were filled with only two seedlings, originating from the seed orchards in Trebon and Plasy. To prevent the overlapping of three individuals within the pot, skewers were used to divert overlapping seedlings. The pots were filled with a mixture of 4.1 kg of Profi-substrat (Gramoflor GmbH & Co. KG, Campemoor 2, Germany) and river sand (1:1 ratio). Plants were acclimated in semi-controlled greenhouse conditions for one week. Cultivation took place over 164 days (from February 21 to August 3, 2022) in semi-controlled greenhouse conditions. After the first 44 days of acclimation (from February 21 to April 7), 405 individuals underwent 84 days of drought treatment (from April 8 to June 30) followed by 36 days of rewatering (from July 1 to August 3), the remaining 387 individuals served as a control in otherwise identical conditions.

The pots were repeatedly, each week loaded onto a transport disk of the PlantScreen™ Modular System (PSI, Drasov, Czech Republic), a high-throughput phenotyping platform in semi-controlled greenhouse conditions located at the PSI Research Centre in Drasov, Czech Republic.

For 164 days of monitored cultivation, a broad range of morpho-physiological traits was regularly measured through kinetic imaging of chlorophyll fluorescence, thermal imaging, top and a multiple angle side view RGB imaging and VNIR/SWIR hyperspectral imaging. For more experimental details and preliminary results on the genetic variability of the photosynthetic traits, see the previous study^30^. In this study, we focused solely on the analysis of hyperspectral image-based data from a VNIR camera.

HSI from three-time points has been analyzed: (1) March 23 (29 days after transplantation - DAT 29), 15 days before water reduction, and Soil Relative Water Content (SRWC) equal to 50%; then (2) June 25 (DAT 123), after the permanent wilting point, and SRWC equal to 0% and finally (3) after the recovery phase on July 30 (DAT 158), with SRWC equal 50%.

### Hyperspectral imaging

Hyperspectral images of Scots pine seedlings were acquired using the HC-900 Series visible (VIS) and near-infrared (NIR) hyperspectral scanner (PSI, Drasov, Czech Republic)^35^. Hyperspectral data were acquired in the 350–900 nm spectral range with a spectral resolution of 0.8 nm full width at half maximum (FWHM) and a spatial resolution of approximately 1.03 mm^2^/pixel. The scanner was mounted on a robotic arm inside a light-insulated imaging box equipped with a 600 W halogen tube light source for homogeneous illumination. The distance between the scanner and plant samples was adjusted for each pot to reach a constant spatial resolution at average plant height. At the beginning of the scanning day, dark current (DC) and white reference (WR) images were acquired. Then plant samples were scanned a sequence lasting no longer than 11 h with sample order randomized every scanning day. Blue mats (Friedola, Germany) were placed on the surface of the pots containing the planted seedlings and blue paint was applied on the planters and skewers to enhance automatic image segmentation of the plant pixels from the background.

The raw data were automatically stored in PlantScreen™ Database (PSI, Drasov, Czech Republic).

### Hyperspectral image preprocessing

The raw images produced by the hyperspectral scanner consist of binary data files accompanied by proprietary text header files. First, the proprietary header file was converted to an ENVI-style header file to enable interaction with the data using commonly used software and libraries. In the second step, reflectance was calculated from the raw data using the formula:

1 $${R}_{\lambda\:}=\frac{{raw}_{\lambda\:}-{DC}_{\lambda\:}}{{WR}_{\lambda\:}-{DC}_{\lambda}}$$

The noisy bands in the range of 350–450 nm were omitted from the subsequent analysis.

### Hyperspectral image processing

To obtain an average spectrum for each plant, the image of each pot was divided into three fixed sections based on the plant position, allowing each plant to be processed separately. Our hyperspectral image processing involved two steps: pre-segmentation and segmentation. The resulting average spectrum for each seedling was then utilized in the treatment and ecotype classification methods: linear discriminant analysis (LDA) and random forest (RF).

### Pre-segmentation

Pre-segmentation was applied to mask out areas that were not plant material, with the goal of reducing background and non-relevant regions. This approach helped to increase the speed and efficiency of the subsequent segmentation process by focusing the analysis on the seedlings themselves. Pre-segmentation of the Scots pine needle HSI was performed using a modified vegetation index (VI), the Modified Chlorophyll Absorption in Reflectance Index 1 (MCARI1)^36^ used in^37^:

2 $$1.2 \times (2.5 \times (R_{{740}} - R_{{672}} ) - 1.3 \times (R_{{740}} - R_{{556}} ))$$

with a threshold level of 0.15, which is a specific cutoff value applied to the calculated MCARI1 values to distinguish between plant material and the background in the hyperspectral images. This VI and threshold segmentation were applied to the entire hyperspectral images, to initially separate the plant regions from the background, creating a first mask to separate most of the background.

MCARI1 was selected for pre-segmentation due to its high sensitivity to chlorophyll variation and strong discrimination capabilities for vegetation pixels^36^. The primary purpose of this pre-segmentation step is to exclude obvious non-plant pixels to reduce computational load. A threshold of 0.15 was selected empirically to be deliberately inclusive rather than restrictive. While MCARI1 can introduce excessive variance with different soil types and non-photosynthetic backgrounds^36^, empirical testing confirmed its suitability for our setup with constant soil and illumination conditions.

### Segmentation

Our primary goal was to develop a hyperspectral data processing pipeline to segment and separate individual coniferous seedlings from the background, allowing for the extraction of their respective spectral means. To achieve this, a segmentation method was applied that accurately differentiates plant material from the background. This process involved using the K-means algorithm, which facilitates clustering of pixels with spectral data into distinct groups corresponding to the plant and background regions. The analysis was conducted using R software^38^, specifically leveraging the “kmeans” function from the “stats” package, with implemented Lloyd’s method^39^ for clustering.

**Fig. 1:**
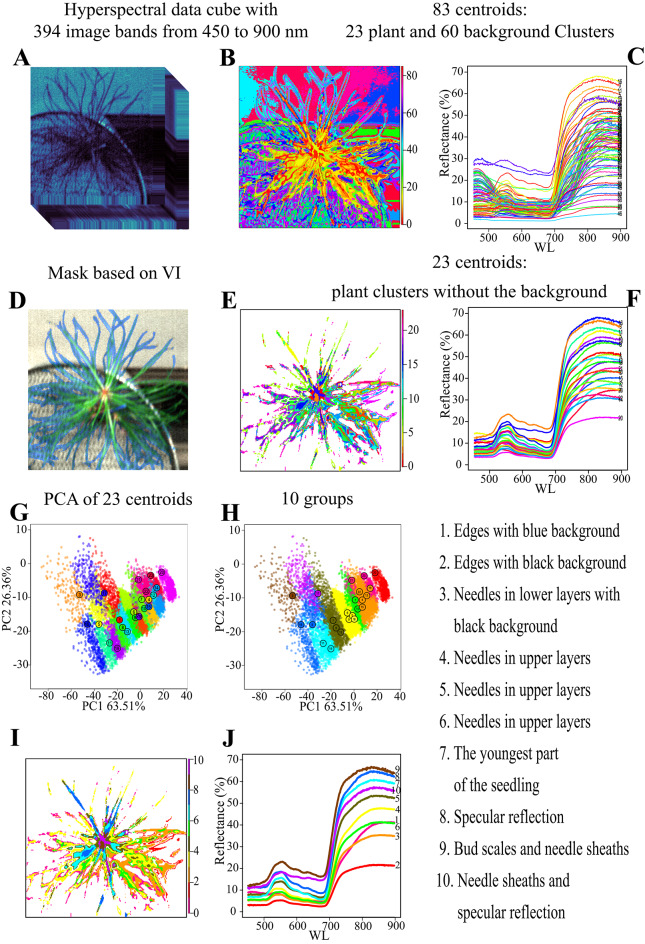
Scheme of the hyperspectral images’ segmentation. (**A**) A representation of a hyperspectral data cube with 394 image bands from 450 to 900 nm. (**B**) Hyperspectral image clustered for 83 clusters by K-means. (**C**) Centroids from the 83 clusters represent reflectance from 450 to 900 nm in 394 bands. (**D**) Mask based on VI with 0.15 threshold. (**E**) Hyperspectral image masked by VI + 0.15 threshold clustered and applied 83 clusters (centroids from (**C**)) by K-means. (**F**) 23 centroids represented plant clusters without the background clusters. (**G**) PCA of the 23 centroids. (**H**) 23 centroids consolidate into 10 groups. (**I**) Region of the interested (seedling) split into ten groups. (**J**) Generalized reflectance patterns of the ten centroids, representing: 1- edges with a black background; 2-edges with the blue background; 3- needles in lower layers with a black background; 4,5,6- needles in upper layers; 7- the youngest part of the seedling; 8- specular reflection; 9- bud scales and needle sheaths; 10- Needle sheaths and specular reflection.

K-means clustering was performed on two non-pre-segmented pots containing six plant images (Fig. 1A), (control and treated pot processed separately for each plant image), taken during the stress period on June 25 (DAT 123), when the variability between controlled and treated plants was the greatest. K-means found natural grouping in the data, generated clusters (Fig. 1B), from which the 83 centroids were manually annotated as representatives of clusters of interest (e.g., Scots pine seedlings 23 and non-pine 60 clusters), (Fig. 1C).

These 83 centroids were then used as initial centers for K-means clustering on all subsequent pre-segmented hyperspectral images (Fig. 1D) and then iteratively updated them for each new image. and used to remove all the pixels belonging to the 60 clusters corresponding to the background. Only the pixels belonging to the 23 plant clusters were left (Fig. 1E) with their plant centroids (Fig. 1F). This means that the K-means clustering process was initialized using the 83 pre-defined centroids, bypassing the usual random initialization step. The algorithm then proceeded directly from these fixed starting points and was run for the default 10 iterations of the kmeans() function in R (since no iteration limit was manually specified). A single pre-trained model was used with the same 83 centroids across all treatments and scanning events to identify pixels with clear vegetation spectral characteristics.

The plant regions clustered based on the remaining 23 plant centroids were analyzed using PCA (Fig. 1G). Based on image inspection and the PCA results, the closest clusters were assigned into ten groups (Fig. 1H) and characterized according to their positions (Fig. 1I) and biological interpretation (Fig. 1J).

### Classification analysis

For further analyses, all plant spectra were averaged giving a single value per seedling. Two classification algorithms, linear discriminant analysis (LDA) and random forest (RF), were employed to differentiate Scots pine seedlings based on their origin during various water stress periods (Table 1). The classification algorithms were always applied to the whole dataset. Statistical analyses were performed in R statistical software^38^.

For LDA “lda” function in “MASS” package was used^40^. The dataset was automatically split into 80% training data (658 samples) and 20% test data (139 samples). The features were standardized using the “preProcess” function in the “caret” package. An LDA model was fitted to predict the locally adapted ecotype and treatment. Predictions on the test set were obtained using the “predict” function. The accuracy of the model was calculated by comparing the predicted classes to the actual class labels in the test set.

For the RF the “RandomForest” package was used^41^. Due to the smaller number of the seedlings from Decin orchard, the weights for the Decin, Plasy, Trebon were adjusted in the ratio of the number of the seedlings using the “classwt” argument. The spectral data comprised 394 wavelength bands ranging from 450 nm to 900 nm. In the RF model, the number of variables randomly sampled as candidates at each split was set to the square root of the total number of variables (√394 ≈ 20). The sample size for training each tree was determined using the default bootstrap sampling method, where approximately 63.2% of the samples were used for training and the remaining samples were utilized for out-of-bag (OOB) error estimation. A total of 1000 trees were “grown in the forest” to ensure robust classification performance. This approach leverages OOB samples to provide an unbiased estimate of the model’s performance without the need for a separate validation dataset.

Model performance was evaluated using confusion matrices generated with the “confusionMatrix” function from “carret” package in R. Cohen’s kappa was computed from each matrix as a chance-corrected measure of agreement, and F1 scores were calculated as the harmonic mean of precision and recall for each class, with macro-F1 obtained as their unweighted average. For the LDA model, these statistics were based on predictions from a 80:20 train–test split, whereas for the Random Forest model they were derived from out-of-bag (OOB) predictions; therefore, the two confusion matrices differ in their absolute values.

## Results

### Generalized reflectance patterns within the seedling

The 23 clusters of reflectance centroids were assigned into 10 groups (Fig. 1F, H) with reflectance patterns determined by seedling position, biological significance, or sensing artefacts (Fig. 1I). Seedling edges, without the background of other needles on black and blue backgrounds, formed groups 1 and 2. These groups showed a very flat reflectance curve in the VIS spectrum, with maximal values reaching only 10%, and 20–40% reflectance in the NIR region.

Needles in lower layers on a black background (group 3) appeared darker green, typical of the previous year’s growth, with 10% reflectance in VIS and 35% in NIR. The reduced reflectance in the blue (450 nm) and red (680 nm) regions is consistent with stronger chlorophyll absorption at these characteristic wavelengths^42,43^ Younger needles from the current year in upper layers (groups 4, 5, and 6) were lighter green, exhibiting a typical plant reflectance curve with absorption maxima in blue and red regions, a peak in the green region (up to 20%), and up to 40–50% reflectance in NIR. The youngest part of the seedling (group 7) had a similar VIS pattern but showed even higher NIR reflectance (up to 60%). In the apical (youngest) part of the seedling, the needles of lower insertion contribute significantly to high NIR reflectance. Additionally, these youngest needles are closer to the sensor and the light source, resulting in a stronger signal. In group 7 — the youngest part of the seedling, the lower layers of the needle significantly increased NIR reflectance. Their proximity to the sensor and light source further amplifies the signal.

Groups 8 and 9 were attributed largely to the specular reflection with the highest VIS reflectance for group 9. In groups 8 and 9, a green peak over 20% and up to 60% was observed in NIR. Group 9 corresponds to the reflection from bud scales and partially the needle sheaths with structures lacking chlorophyll (high reflectance in red). Group 10, representing mostly the needle sheaths and pale needle bases (possibly with specular reflection) exhibited a unique pattern with weak absorption maximum in blue and red regions. Instead, reflectance in the red region was as high as the green peak (almost 20%). In NIR, the bud’s reflectance was lower than groups 8, 9, and 7, but higher than groups 1–6.

### Performance of classification methods

**Fig. 2:**
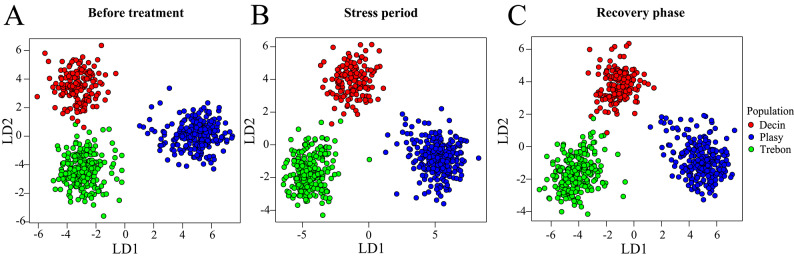
Performance of the linear discriminant analysis (LDA) model in classifying Scots pine seedlings by population origin during different water stress periods. (**A**) represents the beginning of the experiment, (**B**) corresponds to the stress period, and (**C**) shows the recovery phase. The colour dots indicate the following locally adapted populations: Decin (red, upland ecotype) and lowland ecotype: Plasy (blue), and Trebon (green).

The population origin was classified regardless of the irrigation treatment, which was classified regardless of the population origin. The LDA model achieved a prediction accuracy of 80% for the population origin before the treatment, 78% during the stress period, and 81% during the recovery phase, corresponding to error rates of 20%, 22%, and 19%, respectively. Across the three measurement periods, the confusion matrices showed consistent classification patterns among orchards, with misclassification rates closely matching the overall error estimates. The LDA models also yielded moderate to substantial agreement, with Kappa values of 0.699, 0.669, and 0.706, and macro-average F1 scores of 0.791, 0.775, and 0.791 for the pre-treatment, stress, and recovery phases, respectively. For the recognition of the irrigation treatment, the model achieved a prediction accuracy of 88% for the stress period and 77% for the recovery phase, with error rates of12% and 23% for the stress and recovery periods, respectively, (Fig. 2A–C).The confusion matrix for treatment estimation revealed weak agreement before treatment (Kappa = 0.076, F1 macro = 0.537), but classification performance markedly improved during the stress period (Kappa = 0.757, F1 macro = 0.879) and remained moderate during the recovery phase (Kappa = 0.538, F1 macro = 0.769). These results reflect the increasing separability between drought-stressed and control plants once the stress was imposed (Table 2).

In the random forest (RF) model, error rates were higher than in the LDA: for population origin classification with 31% before the treatment, 38% during the stress period, and 38% during the recovery phase. In contrast to LDA, the RF model exhibited reversed pattern of higher accuracy for the population origin before the treatment (69%) and lower predicted accuracy for the population origin (62% for the stress period, and 62% for the recovery period). The confusion matrix (Table 2) of the RF model shows consistent trends with similar accuracy levels for population origin classification across different water stress periods (before treatment, stress period, and the recovery phase). The RF model performed the best in correctly classifying the Trebon population, followed by the Plasy population. However, the Decin population was frequently misclassified, with a significant number of samples being incorrectly identified as Plasy. This misclassification led to higher classification errors for Decin (particularly during the recovery phase this error was up to 65%). The RF model exhibited lower accuracy for population origin classification, with Kappa values indicating only moderate agreement (0.409–0.520) indicating agreement clearly better than random chance but with considerable misclassification, and F1 macro scores ranging from 0.588 to 0.658 across all periods, confirmed moderate overall performance, with the model performing best before treatment initiation (Table 2).

For treatment classification, the RF-predicted accuracy was 54% before the treatment, 83% for the stress period, and 79% for the recovery period) and model estimated error rates of 467%, 17% and 21% before the treatment, for the stress and recovery periods, respectively. The confusion matrix for the RF classification of controlled and treated plants indicates that before the reduction of irrigation, the model struggled to distinguish between the two groups, resulting in similar classification errors (C.E.) for both controlled and treated plants. However, during the period of high stress and the subsequent recovery phase, the model’s ability to correctly classify the plants improved significantly. The C.E. was lowest during the stress period, reflecting the model’s enhanced accuracy under water stress conditions. For treatment classification, the RF model showed negligible agreement before treatment (Kappa = 0.08), substantially improving to strong agreement during the stress period (Kappa = 0.67) and moderate agreement during recovery (Kappa = 0.57), reflecting the model’s enhanced ability to distinguish stressed plants when physiological differences were most pronounced (Table 2).

**Table 1:** Predicted accuracy of classification methods (LDA and RF) for Scots pine seedlings by population origin and treatment across different water stress periods (before treatment, stress period and recovery phase).

Classification method		Before treatment (%)	Stress period (%)	Recovery phase (%)
LDA predicted accuracy	Population	80	78	81
	Treatment	54	88	77
RF predicted accuracy	Population	69	62	62
	Treatment	54	83	79

**Table 2:** Confusion matrix for linear discriminant analysis and random forest classification of Scots pine seedlings by population origin and treatment across water stress periods (before treatment, stress period and recovery phase kappa values indicate agreement beyond chance, classification errors (C.E.) represent misclassification rates, and F1 scores reflect the balance between precision and recall for each class. D, P, T represent population origin (Decin, Plasy, Trebon) and C and D represent control and drought plants. F1 macro represents the average F1 score across all classes.

Linear discriminant analysis (LDA)															
	Before treatment					Stress period					Recovery phase				
*Population*	Kappa: 0.699 F1 macro: 0.791					Kappa: 0.669 F1 macro: 0.775					Kappa: 0.706 F1 macro: 0.791				
	D	P	T	C.E.	F1	D	P	T	C.E.	F1	D	P	T	C.E.	F1
Decin	29	10	6	0.35	0.69	33	13	5	0.35	0.70	26	10	4	0.35	0.66
Plasy	8	49	4	0.20	0.80	7	42	3	0.19	0.76	10	50	3	0.21	0.82
Trebon	2	2	51	0.07	0.88	3	4	49	0.13	0.87	3	1	54	0.07	0.91
*Treatment*	Kappa: 0.076 F1 macro: 0.537					Kappa: 0.757 F1 macro: 0.879					Kappa: 0.538 F1 macro: 0.769				
	C	D	C.E.	F1		C	D	C.E.	F1		C	D	C.E.	F1	
Control	37	34	0.48	0.50		60	11	0.16	0.86		56	16	0.22	0.75	
Drought	40	50	0.44	0.58		8	80	0.09	0.89		21	68	0.24	0.79	
Random forest (RF)															
*Population*	Kappa: 0.520 F1 macro: 0.658					Kappa: 0.409 F1 macro: 0.588					Kappa: 0.418 F1 macro: 0.593				
	D	P	T	C.E.	F1	D	P	T	C.E.	F1	D	P	T	C.E.	F1
Decin	81	86	30	0.60	0.48	69	111	15	0.65	0.40	69	114	14	0.65	0.40
Plasy	43	232	29	0.24	0.70	66	193	41	0.36	0.58	60	195	49	0.36	0.59
Trebon	19	43	244	0.21	0.80	16	56	230	0.24	0.78	15	51	240	0.22	0.79
*Treatment*	Kappa: 0.080 F1 macro: 0.542					Kappa: 0.668 F1 macro: 0.838					Kappa: 0.573 F1 macro: 0.789				
	C	D	C.E.	F1		C	D	C.E.	F1		C	D	C.E.	F1	
Control	220	183	0.45	0.54		342	58	0.15	0.84		322	81	0.20	0.79	
Drought	188	216	0.47	0.54		74	323	0.19	0.83		91	313	0.22	0.78	

## Discussion

### Classification of seedling spectral characteristics

Using hyperspectral images with high spectral and image resolution, parts of the Scots pine seedlings were categorized into groups similar to those reported for sorgum^31^. For split sorghum hyperspectral images into the organs (panicle, stalk, and leaves), classification methods (LDA) were also used. Our camera’s resolution of 1.03 mm^2^ is finer than Miao’s 3.1 × 3.1 mm (9.61 mm^2^). While Miao’s resolution was adequate for sorghum plant parts, high resolution in our study is critical because the projection area of the needles may be below the camera’s spatial resolution. Classification methods (K-means) ensured accuracy by spectral characteristics. Using a PCA combination, groups were considered based on biological interpretation, spatial arrangement, and seedling morphology, and ten groups were described.

In groups 1 and 2, it is evident that the reflectance of seedling edges varies depending on the background. Needles on the blue background (inside the pot) exhibit higher reflectance in the red part of the VIS spectrum and the NIR region compared to the edges of needles sensed on the black background (overlapped needles outside the pot) (Fig. 1D,H,I).

Even though we interpret some of our reflectance classes as pigment-related—because reduced reflectance in the blue and red regions is consistent with stronger pigment absorption^42^ these patterns can also arise from structural and illumination effects. Therefore, the physiological interpretation of spectral features should be made with caution of overlapping biophysical compounds and structural contributions^44^. As the needle layer increases (group 3–7), reflectance in the NIR region also randomly increases. This trend aligns with the findings of^45] and [46^. Although they observed this leaf-stacking effect in planar leaves (such as maple, poplar, and willow)^47^, reported a similar effect in spruce needles. This observation is also supported by simulated reflectance in the radiative transfer model PROSAIL, which shows that reflectance in the NIR increases with the increasing leaf area index^48^. This effect is particularly pronounced in group 7, representing the youngest part of the seedling, where the lower layers of the needle contribute significantly to high NIR reflectance. Additionally, these youngest needles are closer to the sensor and the light source, resulting in a stronger signal.

The highest NIR reflectance was observed in groups 8 and 9 (over 60%), likely influenced by specular reflection. The specular reflections at the top of the seedling area are visible in the RGB picture, the same as observed^49^, (Fig. 1D). Based on previous observations by^50^, it is considered that these groups do not represent pure specular reflectance, as specular reflectance typically reaches unity percentage. However, the specular component may enhance the overall reflectance from the top needles, resulting in higher reflectance levels^49,51^.

Group 3 represents needles from the previous season, characterized by low reflectance values in the blue and red regions, indicating a high chlorophyll content. Groups 5 and 7 exhibit higher reflectance values in the blue region than group 3 indicating lower chlorophyll content in those younger needles. This observation aligns with findings by^52–54^, who reported lower chlorophyll content in current-year needles than those developed in the previous year.

Additionally, the spectral curve shapes in the VIS region, especially in the red region, for groups 8 and 9 exhibited a similar pattern as a curve in^55^, indicating lower chlorophyll content. The pine needles develop basipetally^56^, i.e., they are less mature with lower chlorophyll content at the needle base compared to its tip. The youngest needle bases probably contribute to the central seedling region concentrated around the terminal bud.

Reflectance in VIS for group 10 can be similarly explained as group 9. This group exhibits minimal red absorption representing bud scales and needle sheaths lacking chlorophyll potentially with the specular reflection. The presence of chlorophyll in the bud scales inner scales was described in^57^. It is known that coniferous buds are pre-formed from shoot apical meristem during the vegetative season, which precedes the bud burst and regular shoot growth the following season. Leaf primordia rich in chloroplasts with chlorophyll are covered by bud scales, the innermost of which are still living. Above them, there is a cover of dead bud scales encapsulating the meristem part^58^. The authors showed that the number of living and dead bud scales can differ depending on the seedling damage. Chlorophyllous meristematic cells in leaf primordia could then determine absorption in the red region. Clear classification of this group could be beneficial for phenological studies, such as determining the onset of bud flushing, as suggested by^59^. As was demonstrated for *Picea abies* (L.) H. Karst. buds, living meristems of greenish colouring can be easily distinguished from dead brown buds which can even be used as a criterion of tree vitality based on the proportion of bud developmental types^60^.

### Water stress classification

While stress detection using hyperspectral data is common, our study successfully detected water stress using the RF classification algorithm, achieving 83% accuracy during the stress period and 79% during the recovery phase, even with a spectral range limited to 900 nm, where water absorption maxima are typically not considered^61,62^. Particularly the spectral curve shape of Group 1 (edges with blue background, Fig. 1I) in red and red-edge resembled the spectral curve shape of dehydrated leaves (10% relative water content)^63^, which was probably caused by dehydration and senescence of previous-year needles at the seedling base.

^64^ demonstrated that decreasing water content also affects the visible spectrum, supporting that water stress is detectable within our spectral range (380–900 nm), thus enabling accurate classification of the treated and control plants in the current study. Similarly, red-edge reflectance (734 nm) significantly contributed to the PLSR water content retrieval in Norway spruce needles^54^.

### Genetic variability discrimination by classification algorithms

This study evaluated population genetic variability using classification methods (LDA and RF) based on summary hyperspectral information extracted from canopy-level images of each seedling. The accuracy of the RF variables from the entire 350–2500 nm, acquired using a bare optical cable at the canopy level, was compared for a larger number of the same seedlings measured in the previous season^25^. In the current study, each forest in the RF algorithm used a randomly selected subset of variables (√394 = 20) from our dataset. In the previous study by^25^, the RF accuracy for correctly assigning true origins ranged from 71% to 83%. Our RF accuracy was slightly lower (from 62% to 69%). The frequent misclassification of the Decin population, particularly as Plasy, likely results from the smaller sample size of Decin seedlings used in the experiment. Earlier work by Stejskal et al.^25^ also reported lower representation of Decin seedlings, which could reduce model performance due to class imbalance. In addition, recent study from the same research group^30^ indicate that the genetic variation (heritability) of certain physiological traits (which strongly influence optical properties) is moderate to low, possibly due to the limited geographical gradient of the populations. Moreover, drought stress and recovery may reduce or mask the expression of provenance-specific differences, further decreasing spectral distinctiveness and contributing to misclassification. Despite attempts to compensate for this imbalance by using the “classwt” argument in the RF model to weight the populations, the lower representation of Decin may still have affected the model’s ability to accurately distinguish this group, resulting in higher classification errors.

In contrast, using LDA, similar accuracy to the RF model reported in^25^ (from 78% to 81%) was achieved for experiments conducted before the treatment, during the stress period and in the recovery phase.

The major advantage of non-image hyperspectral data is that it is less labour-intensive in data processing. Moreover, their spectral range could be wider than those provided by hyperspectral cameras, and it might give a spectral response of other plant features, such as water content^61,64^. However, non-image sensors also include background spectra which yields less specific information. Therefore, while non-image hyperspectral sensing can be sufficient for classifying seedling origin, it does not allow for selecting specific seedling morphological components based on their spectral response.

^25^ thoroughly discussed genetic variability assessment using Scots pine needle and seedling level spectral methods. In this study, their findings related to genetic variability were confirmed using canopy-level hyperspectral images.

The PLS-DA models from the non-image hyperspectral data were used to classify fine-scale population structure in two hybridizing Dryas species with an average overall accuracy over 80%^65^. Similar to the classification methods, the regression model used by^24^ also included the entire spectral information (400–2400 nm) to predict the probability of genetic clusters for *Fagus sylvatica* individuals in the forest. This method employs VIP scores to indicate the importance of each wavelength separately. In contrast, previous studies applied linear models that did not incorporate the entire spectral curve simultaneously. Instead, they independently evaluated variability for each wavelength, as demonstrated in *A. thaliana* genotypes^66^. This approach was also applied by^25^, where modified F-test were used to differentiate between populations. Most frequently, spectral index-based detections of genetic variability have been made. For example^67^, compared variability in hyperspectral images within leaf positions between two maize genotypes across three selected wavelengths (498, 550, 625 nm). Similarly^16^, calculated VIs and compared the population means in *P. halapensis* at the vegetation level. Thus, the information from the whole spectral curve was also not utilized.

### Comparative methods for segmentation of conifer seedlings

Plant organs with a small projection area present a greater challenge for segmentation^68^ compared to entire planar leaves, so a comparison of the current study to planar leaf segmentation methods is less relevant as it deals mostly with the segmentation of vegetation patches with small projection areas.

It was necessary to split the picture of the pot into three sections to reduce the computational intensity of the image processing. Unlike^22^, who detected each stem and used a parallelogram-shaped region for the detection of individual positions. Subsequently, they conducted a series of morphological operations to determine stem orientation. Each image was partitioned based on the stable position of the seedling in the pot, with minimal overlap.

Similar to our study, ^22^ validated the hyperspectral imaging approach for detecting the stress tolerance phenotype in loblolly pine seedlings. Similarly^20^, used hyperspectral imaging in the 870 to 1720 nm wavelength range to successfully estimate water content in Chinese fir under simulated drought conditions, further confirming the potential of this approach for detecting stress in forest species. Additionally^23^, used hyperspectral *Pinus* seedlings images to detect nutrient content. However, they mention a threshold value of 0.21 and more details on segmentation process are lacking. It is in agreed with^22^ that a thorough description of the segmentation process is essential for this type of study. The segmentation process is usually done using thresholding based on VIs. While the method also relies on VIs and thresholding (pre-segmentation section), in is considered that this approach may not be sufficient for a comprehensive analysis including detailed biological interpretation. While^19,22^ used VI based on two wavelengths (490 nm, 775 nm and 705 nm, 750 nm, respectively), VI based on three wavelengths (740 nm, 672 nm, and 556 nm) were utilized for thresholding the image. Standard vegetation indices have successfully identified loblolly pine attacked by fusiform rust^19^. However, researchers from the same lab found that using standard indices for segmenting loblolly pine was ineffective in freeze-treated plants due to the mixed colors, prompting them to propose a customized index that considers spectral background information^22^. Although VI was also used for pre-segmenting individual seedlings, the particular VI developed by ^22^, based on specific image series, may have limited applicability in other studies with different species or growth conditions.

For classifying stressed plants^22^, built several standard normal variate discriminant models for each scanning event. In contrast, our study applied the same 23 centroids in the K-means algorithm to all measuring time points (before, during, and after drought stress recovery). Several classification methods (including LDA and RF) were also successfully used for segmenting sorghum (leaf, stalk, tassel/panicle) from hyperspectral images, as demonstrated by^31^.

In ^19^, the faster region-based convolutional neural network (Faster RCNN) architecture^69^ was used for object detection, with complex data processing due to the lack of control over their environment and data acquisition. This complexity aimed to increasing throughput by capturing images of multiple plants together under varying lighting conditions. In contrast, our study ensured high throughput with well-prepared samples and strictly controlled environmental and acquisition conditions without requiring hundreds of annotated images for model training. This additional precision allowed us to avoid the trade-off between data acquisition throughput and processing complexity, achieving efficient and reliable results without excessive data processing.

We believe that our approach preserves data integrity while providing reliable segmentation performance. Pre-trained K-means centroids provide deterministic, reproducible mapping across all scans and time points, which is essential in high-throughput longitudinal settings. The use of VI-based pre-segmentation further reduces the number of pixels analyzed, and the K-means classifier has proven robust enough to compensate for slight variations in illumination without compromising segmentation quality. This approach preserves sufficient data integrity without requiring more complex normalization or supervised learning approaches. The integrity of spectral data can also be affected by changes in illumination or imaging geometry; however, in our case, these distortions were minimized because imaging was performed on an automated transport system with fixed camera orientation and stable artificial illumination, which prevented angle-dependent or illumination-induced shifts in spectral response.

### Growing conditions and geographic origin

Similar to our study, Lu et al. ^22^ measured seedlings in a greenhouse and acquired hyperspectral images of loblolly pine in situ. However, while upland and lowland ecotypes from the Czech Republic were studied, 95 families representing a wide range of provenances, distinguished by various minimal winter temperatures from Florida in the south to Virginia in the north, were used in their study. A more detailed discussion of the growing conditions and geographic origin of our seedlings is provided in our previous studies^25,30^.

In the studies ^19,22^, multiple plants were imaged together with overlapping to balance data acquisition throughput with the number of individuals imaged. Conversely, our study maintained a consistent setup by positioning three plants per pot, minimizing overlap, with each plant oriented uniformly towards the sensor throughout the season. This approach ensured precise and consistent data acquisition. The study of ^35^ also recognizes the importance of maintaining highly controlled conditions for taking images.

### Application

Precise assessment of seedlings’ origin and deployment of the family with increased productivity is used in advanced breeding programs to increase productivity and maintain stress resistance in climate change. It was demonstrated that phenotypic spectral response could generate centroids for classification. These centroids allow the K-means algorithm to specify and classify demanded phenotypes’ spectral patterns. This approach could improve disease resistance of plants or element concentration assessment from hyperspectral imaging^23,70^.

### Limitations

Due to the use of skewers to prevent seedling overlapping, some individuals were captured at an angle matching their deflection rather than directly from the top. However, such growth irregularities occur under natural conditions as well. Therefore, the method should be developed independently based on the nadir camera positioning.

In contrast, our study involved imaging seedlings from the top at a constant height implied by the phenotyping platform setup, resulting in varying distances between plant parts and the sensor. This variability induced strong changes in absolute reflectance value, which in turn produced artificial variability in non-normalized vegetation indices (like selected MCARI1). This effect is especially problematic in quantitative studies aiming to infer concentrations of biochemically relevant compounds, where both spectral shape and absolute size contain important information. However, in our segmentation approach two factors reduced distance to sensor issue: (1) the relatively small vertical variation in sensor distance among the seedlings, reduced illumination-driven magnitude differences across organs. (2) segmentation tasks are generally less sensitive to slight multiplicative shifts in intensity, because the distinguishing information lies primarily in the spectral shape rather, than in absolute scale. The variable sensor distance also introduces spectral mixing effect at needle edges, where pixels capture a combination of reflectance from both needle tissue and the background. Our spectral clustering approach decreases this issue rather than selecting pixels based on spatial location, we classify them based on their spectral signatures. Future studies could further reduce distance-related variability by applying normalization techniques that are correct intensity differences (e.g., SNW-Standard Normal Variate).

Detection by a trained algorithm (e.g., the Faster RCNN architecture) described by^19^ was unsuitable as they imaged seedlings from the side at a constant distance from the sensor and effectively used RGB channels for training and object detection. The intensity of reflected radiation diminishes with the square of the distance from the radiation source, leading to increased background interference, as mentioned^71^. Such variability increases the signal-to-noise ratio, making segmenting plants from the background difficult. It also complicates the training process and increases the risk of overfitting due to the wider range of spectral characteristics. Furthermore, because the images had insufficient resolution, using RGB channels for training was not feasible.

Spectral data integrity can also be influenced by changes in illumination or viewing geometry; however, in our case these distortions were minimized because imaging was performed on an automated conveyor system with fixed camera orientation and stable artificial lighting, preventing angle-dependent or illumination-driven shifts in spectral response. Another limitation of the current pipeline is its potential blindness to novel biological features not represented in the initial centroids. Although these centroids are effective for our dataset, their features are based on spectral responses of the phenotypes present in the “training” images. When analyzing samples exhibiting emerging disease symptoms, unusual stress responses, or rare phenotypic variants, pixels corresponding to these features would be assigned to the nearest existing centroid, potentially resulting in misclassification or unsegmented areas. This could lead to novel discoveries being overlooked or the health status of plants being misinterpreted, as relying on a fixed set of centroids is inherently limiting. The manual selection of the initial 83 centroids introduces subjectivity. To enhance flexibility when implementing, users should upload customized centroid sets tailored to their specific research context or incorporate anomaly detection modules to flag poorly-fitting pixels that may represent novel features requiring investigation.

Biological systems are dynamic and context-dependent, making perfect transferability of any data-driven model—whether for trait estimation or image segmentation—fundamentally challenging^72^. Without periodic updates or retraining, such models may increasingly fail to capture emerging phenotypes or previously unobserved variation.

Additionally, comparing the spectral classes classified by K-means is not possible because their spectral values are identical to the values of the closest centroids used for the classifications. As a result, the spectral values for all classes are the same for each seedling, differing only in their area. Moreover, the absence of ground truth data for our spectral measurements, as highlighted in the context of HSI by^20^, limits our ability to directly compare the features detected by hyperspectral imaging on our fixed phenotyping platform with actual, manually verified data^73^.

## Conclusion

In this study, hyperspectral imaging technology, segmentation using individual wavelengths, and a classification algorithm were used to distinguish local populations of Scots pine and to assess their condition before, during, and after the drought stress. This novel approach allows the non-destructive assessment of Scots pine seedlings in a fully automated controlled environment.

Our workflow integrates traditional image processing methods with the K-means machine learning classification algorithm to segment seedlings and identify biologically relevant plant parts without the need for pre-trained models or proprietary software. The RF and LDA algorithms were successfully applied to classify three locally adapted populations exposed to drought stress. Our findings supported our initial hypothesis and highlighted that this technique employing HSI within the HTP platform is efficient, user-friendly and computationally viable for discriminating locally adapted populations of conifer seedlings.

Implementing this image acquisition and processing method into automated phenotyping platforms could significantly improve high-throughput phenotyping of Scots pine seedlings and become a key tool for nurseries and conifer seedling breeding programs. A manual for segmenting conifer seedlings from hyperspectral image data is provided in GitHub platform.

## Data Availability

Demonstration sample data and their accompanying descriptions are available in the Zenodo repository (https://doi.org/10.5281/zenodo.17167809).The scripts developed for this study are available on GitHub at: https://github.com/JCepl/Pine-hyperspectral-image-segmentaionComplete experimental data and other materials will be made available to third-party academic researchers upon reasonable request.

## Abbreviations

DAT: Days after transplantation
SRWC: Soil relative water content
HSI: Hyperspectral imaging
VI: Vegetation index
PCA: Principal component analysis
LDA: Linear discriminant analysis
RF: Random forest
